# Effects of Cationic Antimicrobial Peptides on Liquid-Preserved Boar Spermatozoa

**DOI:** 10.1371/journal.pone.0100490

**Published:** 2014-06-18

**Authors:** Martin Schulze, Christof Junkes, Peter Mueller, Stephanie Speck, Karin Ruediger, Margitta Dathe, Karin Mueller

**Affiliations:** 1 Institute for Reproduction of Farm Animals Schönow Inc., Bernau, Brandenburg, Germany; 2 Leibniz Institute for Molecular Pharmacology, Berlin, Germany; 3 Department of Biology, Humboldt-University Berlin, Berlin, Germany; 4 Institute of Animal Hygiene and Veterinary Public Health, University of Leipzig, Leipzig, Sachsen, Germany; 5 Leibniz Institute for Zoo and Wildlife Research, Berlin, Germany; Institut National de la Recherche Agronomique, France

## Abstract

Antibiotics are mandatory additives in semen extenders to control bacterial contamination. The worldwide increase in resistance to conventional antibiotics requires the search for alternatives not only for animal artificial insemination industries, but also for veterinary and human medicine. Cationic antimicrobial peptides are of interest as a novel class of antimicrobial additives for boar semen preservation. The present study investigated effects of two synthetic cyclic hexapeptides (c-WFW, c-WWW) and a synthetic helical magainin II amide derivative (MK5E) on boar sperm during semen storage at 16°C for 4 days. The standard extender, Beltsville Thawing Solution (BTS) containing 250 µg/mL gentamicin (standard), was compared to combinations of BTS with each of the peptides in a split-sample procedure. Examination revealed peptide- and concentration-dependent effects on sperm integrity and motility. Negative effects were more pronounced for MK5E than in hexapeptide-supplemented samples. The cyclic hexapeptides were partly able to stimulate a linear progressive sperm movement. When using low concentrations of cyclic hexapeptides (4 µM c-WFW, 2 µM c-WWW) sperm quality was comparable to the standard extender over the course of preservation. C-WFW-supplemented boar semen resulted in normal fertility rates after AI. In order to investigate the interaction of peptides with the membrane, electron spin resonance spectroscopic measurements were performed using spin-labeled lipids. C-WWW and c-WFW reversibly immobilized an analog of phosphatidylcholine (PC), whereas MK5E caused an irreversible increase of PC mobility. These results suggest testing the antimicrobial efficiency of non-toxic concentrations of selected cyclic hexapeptides as potential candidates to supplement/replace common antibiotics in semen preservation.

## Introduction

Antimicrobial substances are necessary and mandatory for the liquid preservation of boar semen. The worldwide development of resistance to antibiotics, not only in artificial insemination (AI) industries, urges researchers to find alternatives to conventional antibiotics. Thus, in order to combat the increase in bacterial resistance, new agents for new targets have to be developed [1]. For this reason, the last decades have seen an intensification of efforts at producing antimicrobial peptides (AMPs) for application in clinical and veterinary settings [2].

AMPs are polypeptides of up to 100 amino acids with a huge structural diversity. More than 5,500 AMPs are known which are effective against a variety of pathogens [3]. Their common characteristic is an amphipathic structure. The spatial separation of the cationic and hydrophobic constituents is an essential prerequisite for their effective interaction with bacterial membranes. This structural feature enables AMPs to interact with the lipids of asymmetrical bacterial membranes in detergence-similar way [4]. Coulomb interactions favor the binding of peptides to negatively charged head groups of bacterial lipids (phosphatidylglycerol, cardiolipin). Hydrophobic interactions allow the peptides to intrude into the hydrophobic region of the lipid bilayer and, in contrast to conventional antibiotics, to destabilize the bacterial cell membrane directly [5].

For an application of AMPs, it is important that they do have only a minor impact on eukaryotic cells. The binding of AMPs to eukaryotic plasma membranes is suppressed since lipids bearing negatively charged head groups are mainly located on the cytoplasmic leaflet in eukaryotic cells, in contrast to prokaryotic membranes [6]. Moreover, the presence of cholesterol in the plasma membrane of eukaryotic cells reduces their membrane insertion [7]. Despite their reduced activity towards eukaryotic membranes, some AMPs possess spermiostatic and spermicidal activity, among them helical magainin A and G, synthetic analogues of the naturally occurring magainin II. Both have been investigated as contraceptives and showed evidence for a concentration-dependent decrease in sperm motility in various mammal species [8], [9]. Other magainin derivatives are non-toxic to normal eukaryotic cells while keeping their high effectiveness against prokaryotic cells [10].

Another group of peptides, rich in specific amino acids such as arginine, phenylalanine or tryptophan, occur as antimicrobial motifs of natural proteins and are also promising candidates for the development of effective and selective AMPs in clinical application [11]. Amino acid exchange and cyclization of an R- and W-rich hexapeptide lead to the cyclic compounds c-RRWFWR (c-WFW) and c-RRWWWR (c-WWW) that show low susceptibility to enzymatic degradation and high selectivity for Gram-negative bacteria [12]. The risk for immune responses is limited because of their small size. Due to their bacterial selectivity, proteolytic stability and thermodynamic resistance, these compounds are promising key structures for application as peptide antibiotics [13].

This study examined the effect of synthetic AMPs on sperm in liquid boar semen preservation. Preliminary investigations selected three synthetic peptide variants: two cyclic hexapeptides c-WWW and c-WFW [13], [14], as well as one helical magainin II amide derivative MK5E [10]. We analyzed the effect of AMPs on sperm motility, membrane intactness *in vitro*, and the fertilizing potential after AI *in vivo*. Applying electron spin resonance spectroscopy (ESR), the impact of AMPs on membrane dynamics was characterized by measuring their influence on spin-labeled lipid analogs incorporated in the sperm cell membrane.

## Materials and Methods

The experimental protocol was approved by the Ethics Committee of our institution (Reg. G 0336/09). This ethics committee was known as Bioethics Commission of the IFN (Bernau, Germany). Present study is not an animal experiment.

### Synthetic Cationic Antimicrobial Peptides

Peptides were provided by Biosyntan GmbH, Berlin, Germany (Table 1). They were synthesized, purified, and characterized as described elsewhere [10], [13]. The peptides were dissolved in diluted HCL, lyophilized, and stored at −20°C. The day before use, stock solutions of 8 mM c-WFW, 4 mM c-WWW, and 2 mM MK5E were prepared in *A. bidest.* and stored at 4°C.

**Table 1:** Molecular weight (MW) and amino acid sequences of the cyclic (c) hexapeptides (c-WFW and c-WWW) and the magainin derivative (MK5E).

Nomenclature	Amino acid sequences	MW (g/mol)
c-WFW	c(RRWFWR)	989.5
c-WWW	c(RRWWWR)	1027.2
MK5E	Ac-GIGKF IHAVK KWGKT FIGEI AKS-NH_2_	2515.1

alanine (A), arginine (R), glutamic acid (E), glycine (G), histidine (H), isoleucine (I), lysine (K), phenylalanine (F), serine (S), threonine (T), tryptophan (W), and valine (V). The linear peptide, MK5E is N-terminally acetylated (Ac) and C-terminally amidated (NH_2_).

### Sperm Samples

Ejaculates were collected from 28 mature Pietrain boars housed at a commercial AI center. For routine semen production, ejaculates are sampled at scheduled days. At these days we retrieved material for our studies by splitting ejaculates. Semen was collected at 5-day intervals by the gloved-hand technique and the gelatinous fraction was removed using gauze. Within 5 min after collection, sperm concentration was determined (SDM 5, Minitüb, Tiefenbach, Germany). Sperm motility was estimated microscopically and only ejaculates with ≥75% motile sperm were included. The semen was extended in Beltsville Thawing Solution (BTS, without antibiotics; Minitüb) at 32±1°C to obtain 2×10^9^ spermatozoa in 90 mL-aliquots, supplemented as described below (experiment 1 and 2, artificial insemination), and slowly cooled to 16°C over a 5 h period. The diluted semen was transported at 16°C to the laboratory for further storage and the respective analyses.

### Experiment 1

After 24 h of semen storage, 10 mL-aliquots of diluted ejaculates from 12 boars were warmed-up for 5 min at 38°C and incubated for further 5 min with increasing concentrations (5 µM, 10 µM, 20 µM, and 40 µM) of the peptides c-WFW, c-WWW, and MK5E. Peptide concentrations were chosen around minimal inhibitory concentrations (MICs). Peptide MICs were previously determined against *E. coli* DH-5α and *Bacillus subtilis* subsp. *spizizenii* DSM 347 [10], [12], [15]. Computer-assisted sperm motility analysis (CASA) was applied for evaluating concentration-dependent AMP effects on sperm motility. BTS supplemented with 250 µg/mL gentamicin (BTS+G) was used as standard.

### Experiment 2

With regard to the results of experiment 1, aliquots of BTS-diluted ejaculates from nine boars were supplemented with 250 µg/mL gentamicin (standard), 4 µM and 8 µM c-WFW, 2 µM and 4 µM c-WWW, and 1 µM and 2 µM MK5E, respectively. Motility and membrane integrity were analyzed for evaluating AMP effects on sperm cells within the common period of usage for AI (12 h) and after prolonged storage (96 h) at 16°C. After 48 h of storage preserved semen was additionally evaluated in a thermoresistance test (TRT: incubation for 30 min and 300 min at 38°C) to assess sperm longevity at body temperature [16]. All samples in experiment 2 were microscopically evaluated for bacterial contamination.

### Evaluation of Sperm Motility

Sperm motility was assessed using a computer-assisted sperm motility analysis (CASA) system (SpermVision, Minitüb, Germany) equipped with a phase contrast microscope (Olympus CX31) and a heating tray (38°C). The CASA-system was operated as described by Schulze et al. [17]. For CASA measurements after 12 h and 96 h storage, 1.5 mL-aliquots were removed from the stored semen and warmed-up for 10 min at 38°C in a heating block (Labnet AccuBlock HT200, Minitüb, Germany). For TRT after 48 h storage (see above), 10 mL-aliquots were removed from the stored semen. Motility was recorded after an incubation of 30 min and 300 min at 38°C in a water bath (GFL 1002, Burgwedel, Germany).

The following parameters were recorded: progressive motility (%), amplitude of lateral head displacement (ALH, µm), beat cross frequency (BCF, Hz), velocity curved line (VCL, µm/s), velocity average path (VAP, µm/s), velocity straight line (VSL, µm/s), linearity (LIN, as a measure of a curvilinear path, VSL/VCL), and hyperactive, progressively motile spermatozoa (%).

### Evaluation of Sperm Morphology

Semen samples were diluted 1∶6 (vol/vol) in 1% PBS-buffered formalin. A 4 µL-aliquot was transferred to a glass slide and covered. Morphology of the sperm head was evaluated by counting 200 spermatozoa using phase-contrast at a magnification of 800x (Jenaval, Carl Zeiss Jena, Jena, Germany). The percentage of cells having a “normal apical ridge” (NAR) of an intact acrosome were estimated.

### Evaluation of Acrosome and Membrane Integrity

A triple-stain flow cytometric method using propidium iodide (PI, Invitrogen, Karlsruhe, Germany), FITC-labeled peanut agglutinin (FITC-PNA, Sigma, Deisenhofen, Germany), and FITC-labeled *Pisum sativum* agglutinin (FITC-PSA, Sigma) was applied to discriminate between viable and dead sperm cells, and to characterize membrane integrity in the acrosomal region as described previously [16].

### Evaluation of Interactions between Peptides and Sperm Cell Membranes by Electron Spin Resonance (ESR) Spectroscopy

The spin labeled (SL) lipids 1-palmitoyl-2-(4-doxylpentanoyl)-phoshatidylcholine (SL-PC), -phosphatidylserine (SL-PS), and N-(4-doxylpentanoyl)-trans-sphingenyl-1-phosphocholine (SL-SM) were synthesized and purified according to the procedure by Fellmann *et al.* [18]. The analogs (1 mM dissolved in chloroform/methanol (1∶1, v/v)) were stored at −20°C. For the experiments, an aliquot of spin labels (18 µL of the stock) was transferred to a glass tube, dried under nitrogen, vortexed with 50 µL BTS, and stored on ice. After 24 h storage of diluted boar semen, spermatozoa were centrifuged twice (6 min, 750×g and 1000×g in an Eppendorf tube, 22°C) and the pellet (about 2.4×10^8^ cells) was resuspended in 150 µL BTS without antibiotic. Sperm cells (50 µL) were labeled with 10 µL spin label solution for 5 min at 22°C, and subsequently, washed with 1 mL BTS to discard non-incorporated analogs and centrifuged again (6 min, 1000×g). Final label concentration was about 6 mol% of endogenous membrane phospholipids as described [19]. Labeled sperm cells were mixed with 25 µL BTS and incubated with 5 µL gentamicin or 5 µL peptide stock to give final concentrations of 250 µg/mL gentamicin, 4 µM c-WFW, 2 µM c-WWW, and 1 µM MK5E, respectively. Before measurement, sodium hexacyanoferrat (K_3_Fe(CN)_6_; Sigma) was added to the samples at a final concentration of 10 mM to reoxidize reduced label molecules [20]. ESR spectra of incorporated spin labeled lipid analogs were recorded at different temperatures (16, 22, and 38±1°C) using an ECS 106 spectrometer (Bruker, Karlsruhe, Germany) with the following parameters: power 20 mW, modulation amplitude 2 G, scan width 100 G, and 4-fold spectrum accumulation. The spectra of pure label suspensions without cells but with peptides (55 µL BTS, 5 µL label, 5 µL peptide) were also recorded.

In some cases, ESR spectra of spin-labeled lipids contained besides the spectral component of the membrane embedded analog an additional component consisting of three narrow signals. This spectrum is caused by free spin-labeled fatty acids formed upon hydrolysis. The degree of hydrolysis was below 10% in all cases. ESR spectra measured were corrected for hydrolysis by subtracting the spectrum of a pure spin-labeled fatty acid. To quantify mobility of spin-labeled lipids, a rotational correlation time τ_c_ was calculated from the spectra according to the formula τ_c_ = 6.5*10^−10^*dH_0_(√((h_0_/h_−1_)−1)) with dH_0_ being the width of the central line (in G) and h_0_ and h_−1_ being the amplitudes of central and high-field lines, as described previously [21].

### Artificial Insemination

Eighty multiparous crossbred sows (40/40; parity: 3 to 6) were used for the artificial insemination experiment. Each sow was checked for heat twice a day. All sows were artificially inseminated two times (12 h and 24 h after the standing heat), using 2×10^9^ sperm in a volume of 90 mL. The insemination was carried out by the same operator. Semen was collected from seven mature Pietrain boars housed at a commercial AI center, extended as described above (standard group: BTS+G; peptide group: BTS containing 16 µg/mL gentamicin +4 µM c-WFW), and inseminated after 12 h and 24 h storage. To ensure a sufficient antimicrobial activity in the preserved semen, a gentamicin concentration was chosen which corresponded to the two-fold MIC for gentamicin-resistant Enterobacteriaceae [22]. Farrowing rate (%), number of piglets born alive (live), number of piglets born dead (stillborn), and total number of piglets (total) were recorded and analyzed.

### Statistics

For testing significant differences (statistic program written by Prof. Hofer, IZW, Berlin, Germany, personal assignment) in sperm quality between the standard (BTS+G) and the AMP variants at the specified times of 12 h, 48 h, and 96 h in experiment 2, a nonparametric analysis according to *Friedman* (*P*≤0.05, two-sided) was followed by a *post hoc* differentiation for single comparisons according to Conover [23]. In experiment 1, a *post hoc* tendency was tested according to Page [23]. Results are represented as box-plot highest and lowest values (*Whisker*) respectively, as inter-quartiles between quartiles 1 and 3 (box), and as median value. Statistical analysis of fecundity, outcomes achieved by the artificial insemination, was performed by paired *Student’s t*-test. Data are expressed as mean±SD. Significance was defined as *P*≤0.05.

## Results

### Concentration-dependent Short-term Effects of AMPs on Sperm Motility (Experiment 1)

**Figure 1:**
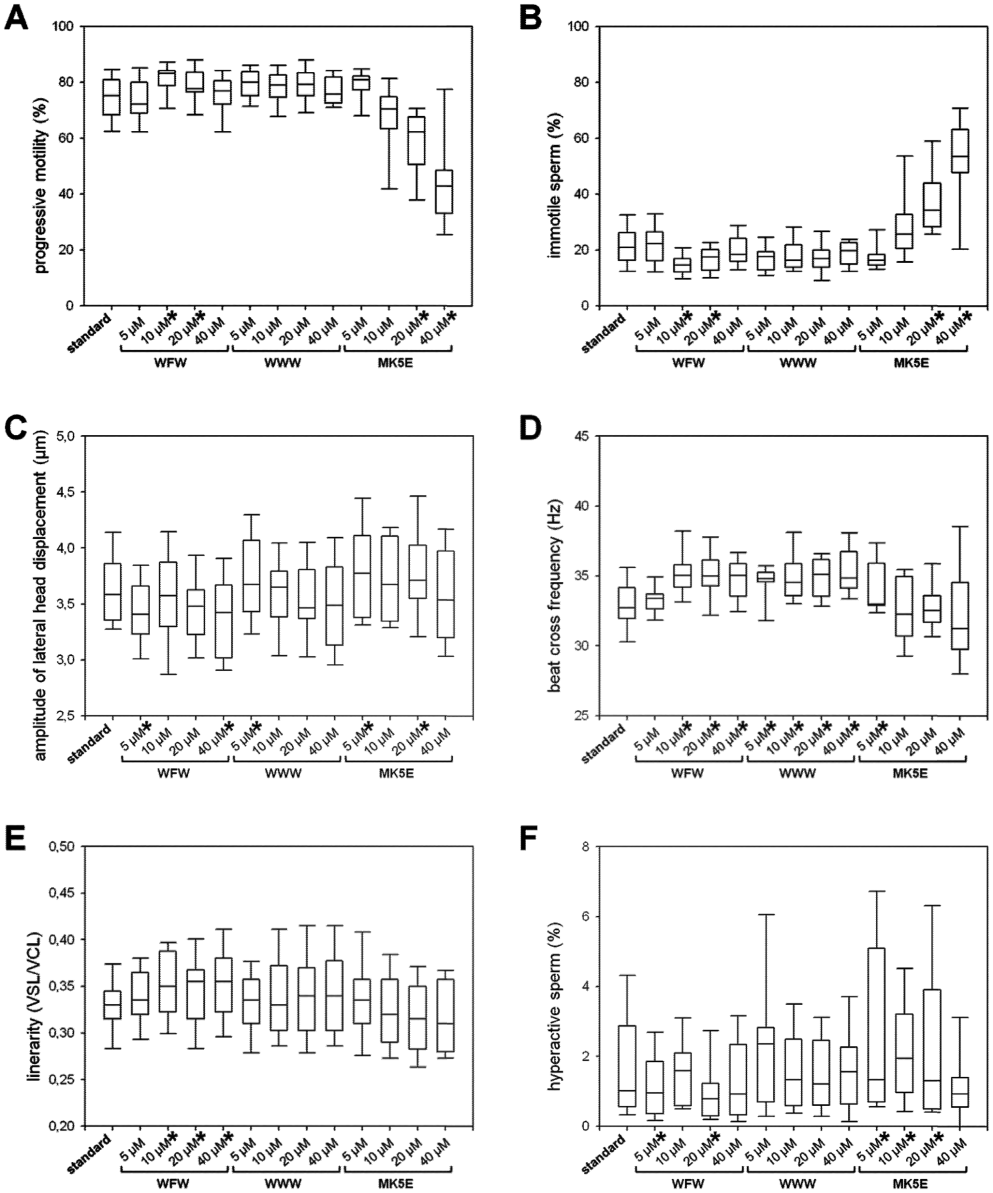
Motion parameters of boar spermatozoa after 5 min of incubation with increasing concentrations (5 µM, 10 µM, 20 µM, and 40 µM) of c-WFW, c-WWW, and MK5E (n = 12). Before the application of peptides, semen was preserved for 24°C in BTS containing 250 µg/mL gentamicin (standard). * Significant difference compared to standard (see text for the exact *P*-values).

To determine concentrations applicable for sperm preservation, AMPs were screened for their short-term effects on sperm motility. After 5 min of incubation at 38°C, c-WFW and MK5E exerted contrary effects on boar sperm motility. Whereas 20 µM and 40 µM MK5E significantly (*P*<0.001) decreased the proportion of progressive motile sperm, c-WFW had no influence or even increased the progressive sperm motion at 10 µM and 20 µM of this hexapeptide (*P*<0.001 and *P* = 0.013, respectively, Figure 1A). Concomitantly, the proportion of immotile sperm was increased by MK5E (*P*<0.001) and decreased by c-WFW (*P*<0.001, Figure 1B) at the respective concentrations. With c-WWW, no significant changes of these parameters were observed.

C-WFW caused a significantly (*P* = 0.003) lower amplitude of lateral sperm head displacement (ALH, Figure 1C), tied to a significantly (*P*<0.001) higher beat cross frequency (BCF, Figure 1D). A significantly (*P*<0.001) higher proportion of linear motile sperm and a higher linearity of progressive motile sperm were consistently observed already at a concentration of 10 µM and beyond (Figure 1E). C-WWW exerted an augmentation of BCF at all tested concentrations (*P*≤0.002) and a decreased ALH at 5 µM (*P* = 0.004).

Incubation of sperm with 5 µM or 20 µM MK5E induced a significant (*P* = 0.017) increase of ALH, a tendency for lower BCF, and a significantly (*P*≤0.029) higher proportion of hyperactive sperm at 5 µM, 10 µM, and 20 µM (Figure 1F). The latter parameter was reduced with 5 µM and 20 µM c-WFW (*P* = 0.02 and 0.028).

### Effects of AMP Supplementation on Sperm Quality during Storage (Experiment 2)

Based on the data of short-term experiment 1, the hexapeptides c-WFW and c-WWW were further tested at concentrations of 4 µM and 8 µM, as well as 2 µM and 4 µM during semen storage. Because of its negative effects on sperm motility, MK5E was only tested at concentrations below 5 µM. Microscopic evaluation of samples did not reveal obvious bacterial contamination in any of the tested variants.

**Figure 2:**
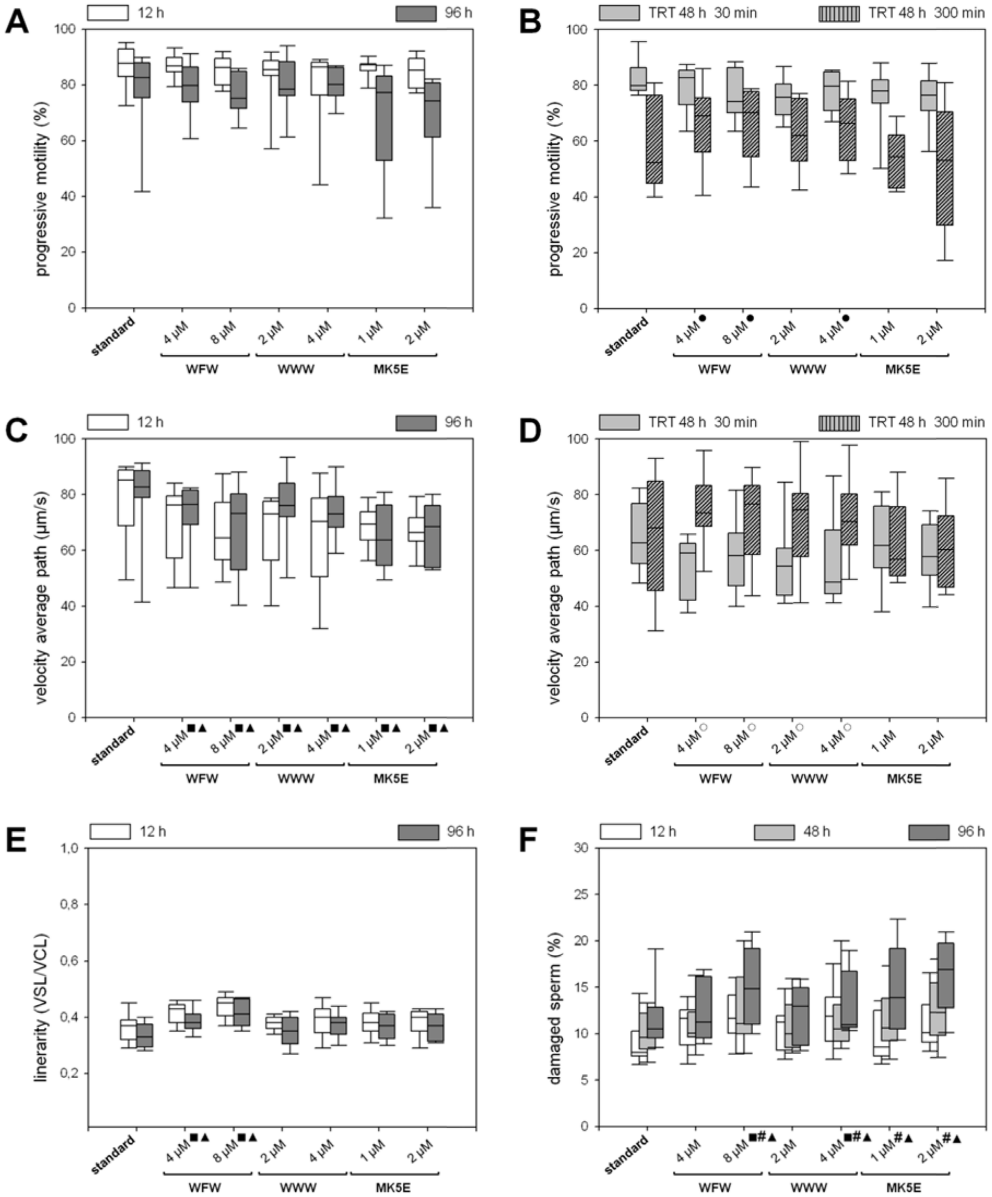
Motion parameters and percentage of damaged boar spermatozoa during BTS preservation at 16°C in the presence of 250 µg/mL gentamicin (standard), 4 µM and 8 µM c-WFW, 2 µM and 4 µM c-WWW, and 1 µM and 2 µM MK5E (n = 9). The percentage, the velocity (VAP), and the linearity (VSL/VCL) of progressive motile spermatozoa after 12 h and 96 h storage are shown (A, C, E). The percentage and the velocity (VAP) of progressive motile spermatozoa after 48 h storage are shown in a thermoresistance test (TRT, incubation at 38°C) for 30 and 300 min (B, D). The percentage of damaged spermatozoa was determined after 12 h, 48 h, and 96 h storage by staining with propidium iodide and/or markers for the acrosomal membrane and contents (F). Significant difference (see text for the exact *P*-values) compared to standard after 12 h (▪), 48 h (#), 96 h (▴) storage, and after TRT 30 min (○) and TRT 300 min (•) incubation at 38°C.

At the time points investigated during storage, no significant peptide effect on the percentage of progressive motile sperm was found (Figure 2A). After 48 h of semen storage, a TRT was used to address the sperm performance in the female genital tract after AI. Particularly after 300 min incubation at 38°C the proportion of progressive motile sperm decreased (Figure 2B). However, the stress-related motility loss after 300 min incubation varied for both peptide groups. C-WFW and c-WWW supplemented samples showed the highest stability with even higher motility values after 300 min incubation than the standard (*P*≤0.009). MK5E (2 µM) caused lowest motility values after 300 min incubation in TRT. Also, the highest proportion of immotile sperm resulted from MK5E supplementation, by contrast, the lowest proportion of immotile sperm at the end of TRT was observed in the hexapeptide variant at 8 µM c-WFW. All AMP supplements reduced VAP of progressive motile sperm in comparison to the standard at 12 h and 96 h storage (*P*≤0.01, Figure 2C). After 48 h and 30 min TRT, this effect was only observed with the hexapeptides (*P*≤0.01, Figure 2D). An extended incubation in TRT for 300 min, led to a recovery of velocity in the hexapeptide-exposed samples compared to the values at the beginning of the test. No recovery of VAP was found for MK5E.

Linearity of progressive sperm motion reflected a significant influence of the hexapeptide c-WFW (Figure 2E). In particular, c-WFW-exposed sperm moved significantly (*P*<0.001) more linear as a function of the concentration level and those samples had more linear motile sperm after 12 h and 96 h storage (*P*<0.001 and *P*≤0.005). MK5E had no effect on linear sperm motility. In accordance with the outcome on linearity, ALH was reduced significantly (*P*≤0.01) by the hexapeptides after 12 h and 96 h storage, and after 48 h at the beginning of the TRT (*P*≤0.005). C-WFW exerted the most pronounced effect. The proportion of hyperactive sperm was significantly reduced below the standard level after 12 h and 96 h storage with hexapeptides (*P*≤0.02 and 0.008).

For visualization of membrane defects, propidium iodide (PI) and fluorescent lectins FITC-PNA and FITC-PSA were applied. Flow cytometry data revealed an overall low rate of damage to membranes and acrosomes by hexapeptides compared to the magainin derivative (Figure 2F). Significance levels were reached for 8 µM c-WFW and 4 µM c-WWW after 12 h, 48 h, and 96 h storage (*P*<0.001). Using MK5E, both concentrations caused a significant (*P*<0.001) increase in damaged sperm after 48 h and 96 h storage. The damaged sperm population consisted of a majority of PI permeable (dead) sperm with concomitant membrane disturbance in the acrosomal region. The microscopically counted proportion of sperm with “normal apical ridge” (NAR) in the presence of the hexapeptides showed no significant difference to the standard. The proportion of sperm with NAR, though, showed a significant (*P*<0.001) decrease after supplementation with 2 µM MK5E. This effect gradually increased with longer storage times (unpublished data).

### Impact of AMPs on Membrane Dynamics

To further characterize the interaction between peptides and the sperm cell membrane, the ESR spectra of various spin-labeled phospholipid analogs (SL-SM, SL-PC, and SL-PS) incorporated into the cell membrane of boar sperm were recorded in the absence (solely BTS) and in the presence of AMPs. These experiments were performed in order to investigate whether AMPs have an impact on membrane dynamics since the shape of ESR spectra reflects the mobility of SL-PL. To quantify this mobility, rotational correlation times (τ_c_) were derived from the ESR spectra (Table 2). Decreasing values correspond to increasing mobility of the spin-labeled lipids and vice versa. As expected, for all labels an increased fluidity was measured with increasing temperature. Comparison of mobility of the different phospholipids showed lower correlation times for SL-PS than for SL-SM, whereas for SL-PC, medium correlation times were estimated.

**Table 2:** Rotational correlation times (τ_c_) for spin-labeled (SL) sphingomyelin (SL-SM), phosphatidylcholine (SL-PC), and phosphatidylserine (SL-PS) incorporated in the cell membrane of fresh boar sperm, recorded in BTS and in the presence of gentamicin, c-WFW, c-WWW, and MK5E at 16°C, 22°C, and 38°C, respectively.

SL-lipid	Temperature	Correlation time τ_c_ (ns)				
				Peptides		
		BTS^a^	Gentamicin^a^	c-WFW^a^	c-WWW^a^	MK5E^a^
SL-SM	16°C	3.94	3.62	4.39	4.42	3.06
	22°C	3.04	n. d.	4.17	3.91	2.83
SL-PC	16°C	2.58	2.56	3.12	3.30	2.41
	22°C	2.61	2.18	3.17	3.06	1.93
	38°C	0.95	n. d.	1.12	n. d.	0.86
SL-PS	16°C	2.32	2.26	2.45	2.44	2.53
	22°C	1.85	n. d.	n. d.	1.87	1.87

*N*-(4-doxylpentanoyl)-*trans*-sphingenyl-1-phosphocholine (SL-SM).

1-Palmitoyl-2-(4-doxylpentanoyl)-phoshatidylcholine (SL-PC).

1-Palmitoyl-2-(4-doxylpentanoyl)-phosphatidylserine (SL-PS).

not determined (n. d.).

a Values are means calculated from spectra of four independent sperm samples (SD<0.05).

In the presence of c-WFW and c-WWW the mobility of the spin-labeled lipids was decreased as seen from the increased correlation times compared with the control samples. This impact was more conspicuous for SL-SM and SL-PC compared to SL-PS. MK5E caused a fluidization of the sperm cell membrane which was recorded for SL-PC and SL-SM, whereas this effect was not observed for SL-PS. A specific interaction between peptides and the spin-labeled lipids could not interfere with our data since each peptide showed a similar interaction with all analogs in aqueous solution, i.e. in BTS without sperm (unpublished data).

For measuring the reversibility of the interaction between peptides and sperm cell membranes, SL-PC and SL-SM-labeled sperm were washed in BTS after measurement, resuspended in BTS without peptides, and measured again. The immobilizing effects of cyclic hexapeptides completely vanished (unpublished data), while the fluidizing effect of MK5E was still present after washing.

### Artificial Insemination with AMP Supplemented Semen

The fertilizing potential of semen preserved in the presence of 4 µM c-WFW and a reduced amount of gentamicin (16 µg/mL) compared to the standard (BTS+G) was not affected (Table 3). Non-pregnant sows returned to oestrus regularly. The lowest concentration of the best suited cyclic peptide had obviously no negative effect on sow fertility.

**Table 3:** Fecundity results with semen diluted in BTS +250 µg/mL gentamicin (standard) and 16 µg/mL gentamicin +4 µM c-WFW (peptide).

	Sows^a^ (n)	Farrowing rate^b^ (%)	Piglets born (mean ± SD)		
			Total^b^	Live^b^	Stillborn^b^
Standard	40	90.0	11.5±4.9	10.2±4.7	1.3±0.7
Peptide	40	87.5	11.7±5.1	10.4±4.8	1.3±0.7

a Two inseminations per sow were performed with 2×10^9^ sperm per dose.

b Differences are not significant (*P*>0.05).

## Discussion

With regard to increasing bacterial resistance against standard antimicrobials (e.g. gentamicin) for boar semen preservation, research on alternative substances is recommended [24]. Besides broad antimicrobial activity, less or no sperm toxicity is prerequisite. Consequently, examination of sperm compatibility is crucial for the development of novel preservatives.

Experiment 1 used CASA for examining short-term effects of increasing concentrations of three synthetic AMPs. AMP effects on sperm motility were clearly concentration-dependent. C-WFW (10 µM, 20 µM) revealed a distinct positive effect on the proportion of progressive motile sperm. It also revealed an increase in the linearity of sperm movement. ALH decreased and concurrently, BCF increased. Hence, c-WFW supplementation advanced linear motility. Supplementation of c-WWW revealed less pronounced short-term effects on boar sperm.

In contrast to the hexapeptides, MK5E (20 µM and 40 µM) exerted a negative effect on motility. This is in accordance with other studies that reported on reduced sperm motility caused by magainins [8]. In our study, the percentage of progressive motile sperm and their velocity VAP decreased, and the number of immotile sperm was increased. Moreover, a higher ALH combined with a lower BCF resulted in a larger proportion of hyperactive sperm at peptide concentrations between 5 µM and 20 µM. Interestingly, this MK5E-effect on boar sperm is similar to that observed in sperm capacitation [25]. The short-term effects on boar sperm caused by AMPs suggest a peptide-specific interaction with eukaryotic membranes. Although in former studies hemolytic activity as well as toxicity against eukaryotic human cells at peptide concentrations up to 200 µM was negligible (c-WWW, c-WFW) [13] to non-existent (MK5E) [10], boar spermatozoa were obviously affected at much lower peptide concentrations.

To characterize peptide-sperm interactions at membrane level, spin-labeled phospholipid analogs (SL-PC, SL-PS, and SL-SM) in conjunction with ESR were used as reporter molecules. Both hexapeptides, c-WFW and c-WWW, caused an increase in correlation times (τ_c_) particularly for SL-SM and SL-PC showing an immobilization of these analogs in the sperm cell membrane. In the presence of MK5E, however, ESR spectra reflected a larger fluidity. Again the effects of the peptide additive on rotation mobility of the spin-labeled phospholipid analogs was far more pronounced for SL-SM and SL-PC than for SL-PS, e.g. fluidizing effects of MK5E on SL-PS were totally absent. The observation correlates with the enhanced permeabilizing effect of MK5E as well as the parent sequence M2a on neutral lipid bilayers compared to negatively charged model membranes [10], [26].

The differences in peptide-analog-interaction can be explained by the preferred transbilayer localization of analogs which is in line with transbilayer (asymmetric) distribution of endogenous phospholipids in the plasma membrane of eukaryotic cells [6]. According to that SM is being located predominantly on the outer and PS mainly on the inner half of the cell membrane of live sperm [27]. General assumption states that fluidity in the membrane half oriented to the cytoplasm is higher than on the exoplasmic side [6]. PC is distributed nearly equally between both membrane halves. Consequently, the ESR studies underline an interaction of peptides, predominantly with lipids on the exoplasmic membrane half.

Vanishing effects of cyclic hexapeptides after washing peptide-treated sperm cells argue for a reversible peptide-membrane interaction. Rigidification of the cell membrane was tied to the presence of these peptides and the affinity to the membrane seemed to be low. This observation is in accordance with a low partition coefficient of cyclic R-, W-rich peptides into lipid matrices, a shallow insertion into negatively charged lipid bilayers [12], [28], and very low lipid bilayer permeabilizing activity [13].

Viewing motility parameters in the light of ESR data, a more rigid sperm cell membrane after incubation with c-WFW might be responsible for the decreased ALH and the resulting increased linearity of the sperm movement since a certain flexibility of the tail membrane is essential for its bending. The mechanical resistance of a more rigid membrane would result in a less vigorous undulating bending of a more elongated tail and, thus, smaller amplitude of head displacement. Instead, the frequency of bending may increase and in turn mediate the more linear movement.

In contrast to cyclic hexapeptides, sperm incubation with MK5E caused a fluidization of membranes. The fluidizing effects of MK5E largely persisted after washing peptide treated sperm cells (irreversibility). Our data can be interpreted as changes similar to capacitation specified by Mortimer *et al.* [29]. Previous studies by other researchers demonstrated the capability of detergent-like molecules to generate a cholesterol efflux from the sperm plasma membrane [30]. Cell membranes of boar sperm already have an extremely low cholesterol/phospholipid (C/P) ratio [31]. This C/P ratio is further reduced by depriving the cell membrane of cholesterol which represents an essential event of capacitation [32]. Changing the composition of lipids can also induce a fluidization and/or under certain conditions a destabilization of the sperm cell membrane [33]. Since AMP molecules may intrude into the hydrophobic region of the lipid bilayer [5], they may influence not only a membrane’s fluidity but also lipid-protein interactions and permeability. Magainin peptides are well known to significantly disturb the lipid arrangement and, e.g. to increase permeability towards polar molecules [10]. With regard to semen preservation, it is desirable that a premature capacitation is prevented. Therefore, concerning all this short-term interaction data, the hexapeptides would be favored over the magainin derivative because they are even capable of promoting a linear sperm movement.

Based on results of experiment 1, lower concentrations of all AMPs were tested for their long-term effects during semen preservation at 16°C. In experiment 2, c-WFW was again able to promote a more linear motion. Also, when stored sperm cells were challenged in a TRT, the supplementation with hexapeptides resulted in a more progressive and linear movement with a higher VAP and lower ALH compared to standard. This stimulation might be advantageous with regard to the further performance of inseminated semen in the female genital tract [17], [34]. Only in the presence of MK5E, the percentage of progressive and linear motile sperm decreased, and that of immotile sperm increased. These effects became more evident after prolonged storage (96 h).

Opposed to de Waal et al. [35], a disruption of mitochondrial activity was not the cause for the spermicidal effect (unpublished data). Morphological examinations revealed a concentration-dependent diminution of the proportion of sperm with “normal apical ridge”, in particular for MK5E. Flow cytometry after staining with PI and fluorescent lectin-markers for membrane defects verified AMP effects and demonstrated higher proportions of membrane-damaged sperm at higher levels of MK5E as well as of c-WWW and c-WFW. However, as to conditions of low concentrations of hexapeptides, loss of *in vitro* vitality compared to the conventional preservation in standard BTS+G remained within arguably pragmatic limits. All in all, hexapeptides induced lower membrane and acrosome damages compared to the magainin derivative.

## Conclusions

The present study suggests that the AMPs tested here interact specifically and differently with the eukaryotic cell membrane of boar spermatozoa. The data revealed that effects due to c-WWW (2 µM) and c-WFW (4 µM) remained within acceptable range for liquid boar semen preservation. Moreover, c-WFW (4 µM) had a positive impact on the proportion of progressive and linear moving sperm, and improves sperm longevity during incubation at 38°C *in vitro*. Also, sow fertility was not negatively affected *in vivo* after AI with boar sperm preserved in the presence of c-WFW (4 µM) and reduced gentamicin (16 µg/mL). Therefore, cyclic hexapeptides, particularly c-WFW, are candidates to be tested for their antimicrobial potential *in situ* as an alternative or supplement to conventional antibiotics in boar semen preservation.

## Acknowledgments

The authors thank Hauke Hönicke for providing boar ejaculates and Anita Retzlaff for her excellent technical assistance in the lab. We thank Prof. Heribert Hofer for the possibility to use his statistics program “Friedman, Quade and Page tests” (Version 2009).
